# Automated analysis of mitochondrial dimensions in mesenchymal stem cells: Current methods and future perspectives

**DOI:** 10.1016/j.heliyon.2023.e12987

**Published:** 2023-01-18

**Authors:** Sabrina Summer, Agnes Kocsis, Eva Ingeborg Reihs, Mario Rothbauer, Kirill Lonhus, Dalibor Stys, Peter Ertl, Michael B. Fischer

**Affiliations:** a Department for Biomedical Research, Center of Experimental Medicine, Danube University Krems, Dr.-Karl-Dorrek-Straße 30, Krems an der Donau, Austria; b Karl Chiari Lab for Orthopaedic Biology & Ludwig Boltzmann Institute for Arthritis and Rehabilitation, Department of Orthopedics and Trauma Surgery, Medical University of Vienna, Währinger Gürtel 18-20, Vienna, Austria; c Institute of Applied Synthetic Chemistry and Institute of Chemical Technologies and Analytics, Technical University Vienna, Faculty of Technical Chemistry, Getreidemarkt 9/163, Vienna, Austria; d Institute of Complex Systems, Faculty of Fisheries and Protection of Waters, South Bohemian Research Center of Aquaculture and Biodiversity of Hydrocenoses, University of South Bohemia in České Budějovice, Zámek 136, 373 33 Nové Hrady, Czech Republic; e Clinic for Blood Group Serology and Transfusion Medicine, Medical University of Vienna, Währinger Gürtel 18-20, Vienna, Austria

**Keywords:** Mitochondrial dynamics, Mesenchymal stem cells, Microfluidic systems, MSC-Based therapies, Automated mitochondrial analysis

## Abstract

As centre of energy production and key regulators of metabolic and cellular signaling pathways, the integrity of mitochondria is essential for mesenchymal stem cell function in tissue regeneration. Alterations in the size, shape and structural organization of mitochondria are correlated with the physiological state of the cell and its environment and could be used as diagnostic biomarkers. Therefore, high-throughput experimental and computational techniques are crucial to ensure adequate correlations between mitochondrial function and disease phenotypes.

The emerge of microfluidic technologies can address the shortcomings of traditional methods to determine mitochondrial dimensions for diagnostic and therapeutic use. This review discusses optical detection methods compatible with microfluidics to measure mitochondrial dynamics and their potential for clinical stem cell research targeting mitochondrial dysfunction.

## 1 Introduction

Microfluidic technologies are promising platforms to monitor cellular functions at a microscale with significantly reduced sample size and increased reproducibility, conducted either on-chip or off-chip. Utilizing single- or multi-cell cultivation-based microfluidics, biological applications, such as cellular phenotyping, cell-cell interactions, and omics profiling, can be performed in real-time under physiological conditions. Bio-fabrication-based technologies for construction of functional cell or tissue replacement models extend the portfolio to manufacture biostructures with different but defined dimensions to generate a controlled microenvironment. Combination with microfluidic devices allows the formation of tissue-like structures with various shapes and bio-mechanical features for microscale analysis of two-dimensional (2D) monocultures and three-dimensional (3D) models, such as spheroids, organoids, and tissue-like constructs [[1], [2], [3]].

Organ-on-a-chip systems that can recapitulate complex organ-organ or tissue-tissue interfaces (e.g., myocardial patches, the blood-brain barrier, the air-blood barrier, and vascularization of decellularized tissue-on-a-chip) extend the possibilities of the use of microfluidic-based technologies to mimic microphysiological systems (MPS) that eventually substitute for animal testing in the future.

Mitochondria are crucial to produce metabolic energy in the cell as well as to regulate key metabolic and cellular signaling pathways [4]. As highly dynamic organelles, they exist in different shapes, ranging from punctuate structures to tubular networks, and their cellular distribution can be quite heterogenous. The number of mitochondria per cell is tightly regulated by mitochondrial biosynthesis and increases under metabolic or environmental stress to maintain their function, making them a crucial indicator for the physiological state of the cell [5,6]. There is growing evidence highlighting the importance of mitochondrial volume homeostasis in critical processes of the cell, where changes coincide with pathologies or pharmacological treatments affecting mitochondrial function [7,8]. This emphasizes the need for high-resolution detection and image processing algorithms of mitochondrial shape, volume, and distribution in mesenchymal stem cells (MSCs), which are widely used as therapeutic agents in inflammatory diseases and a broad range of mitochondrial dysfuntions, including but not restricted to cardiovascular diseases and metabolic disorders [9,10]. Commonly used technologies to determine cellular function are multiplexed flow cytometry and multicolor confocal microscopy. Imaging detection systems produce large data sets as basis of 3-dimensional analyses which are analyzed by accurate image processing algorithms. These algorithms should be able to cover digital image detection, reconstruction, correction, and data compression. There are several open-source algorithms available that are compatible with high-throughput data generation platforms (e.g., microfluidic chambers) which we will discuss in section 5.

This review comprises currently available tools to investigate dynamic changes of mitochondria in response to metabolic changes and environmental stress in MSCs, giving special attention to the use of microfluidic technologies. Limitations and future challenges of automated mitochondrial analysis using high-throughput analysis on microfluidic platforms will be emphasized.

## 2 Processes affecting mitochondrial dimensions

**Fig. 1:**
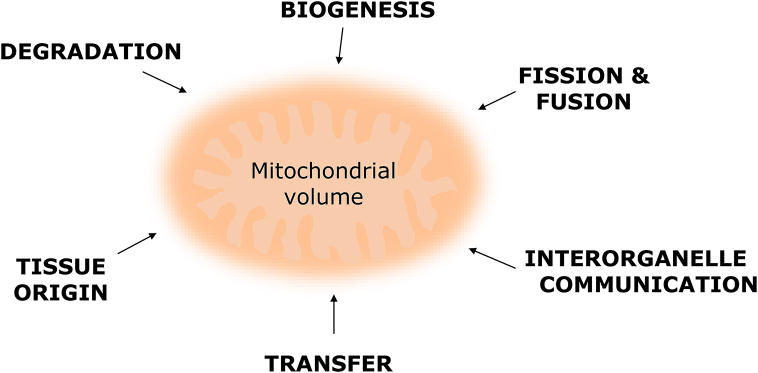
Processes that influence the homeostasis of mitochondrial dimensions.

To understand the complexity behind the determination of size, shape, and structural organization of mitochondria (further referred to as mitochondrial dimensions), the most prominent factors (Fig. 1), including intracellular mechanisms and intercellular communication, which can influence mitochondrial measurements using microfluidic devices are summarized here.

### 2.1 Dynamic tissue microenvironment

Tissue and organ function rely on the ability of tissue-resident cells to sustain energy requirements by producing adenosine-triphosphate (ATP) by oxidative phosphorylation (OXPHOS). Cell-to-cell oxygen supply depends on microvascular perfusion and hemoglobin uptake. Using microfluidic devices, nutrient and oxygen delivery can be controlled preserving the original architecture of the tissue sample. A native tissue-specific extracellular matrix (ECM) can be maintained, which regulates important mechanisms for microenvironmental mechanics, including the Yes-associated protein (YAP) or focal adhesion kinase (FAK) pathway. Alterations in mitochondrial function and dynamics also affect the cytoskeletal machinery [11].

A deficiency in the synthesis of the mitochondrial-encoded subunits of the respiratory chain alters the ECM composition in cartilage tissue, resulting in collagen crosslinking and an increased ECM stiffness [12]. The ability of mitochondria to adapt to mechanical stressors in the microenvironment has also been associated with several pathologies, reviewed elsewhere [[13], [14], [15]]. Organ-on-a-chip or tissue-on-a-chip in combination with microfluidics maintain the integrity of the sample preserving organelle function. Visualization of cellular interaction within living tissue in response to microenvironmental changes can be addressed; however, the accuracy of the outcome depends on the complexity of the designed model. Metabolites, soluble factors, and cell-type specific nutrients need to be determined to sustain the tissue integrity and functionality.

### 2.2 Intracellular events

Mitochondria are highly dynamic organelles and undergo structural modifications due to biogenesis, fusion and fission, and degradation at the lysosomal level (mitophagy), as reviewed elsewhere [[16], [17], [18], [19], [20], [21], [22], [23], [24]]. Within microsystems, such as microfluidic devices, the mechanisms of mitochondrial volume regulation may vary due to exposure to environmental stimuli, e.g., pH, salt concentration, temperature, and mechanical stress, or differences in the biological function of the cell types present [25,26]. The number of mitochondria varies between zero and a few thousands per cell; showing high abundancy in liver and muscle of up to 5000 mitochondria per cell [[27], [28], [29]]. Overall, the balance of the above-mentioned regulatory mechanisms ensures the maintenance of mitochondrial integrity and, consequently, the biological functions of the cell [30,31]. Microfluidic-based lab-on-chip systems enable the supply of multiple cell types within complex culturing systems providing models to mimic the physiology of cells in living tissue. To resemble an adult-like phenotype within these microscale systems, the use of pluripotent stem cells, e.g., MSCs, might be beneficial; even though donor variability and restricted differentiation potential could affect the reproducibility. MSCs aid in the regulation and coordination of cellular metabolism in surrounding cells, mostly through mitochondrial transfer [9,32]. Placenta-derived MSCs can ensure the correct sequence of essential processes in the organism, such as trophoblast invasion during embryo implantation, by controlling mitochondrial dynamics by regulating key players of the mitophagy pathway [32,33]. An impairment of these regulatory events results in morphological changes giving rise to elongated, interconnected mitochondrial system or to fragmented, discontinuous mitochondria [34].

Mitochondrial homeostasis and the regulation of their precise localization involve three types of motor proteins that translocate along cytoskeletal tracks, myosin moving along actin filaments, and kinesin, and dynein moving along microtubules (MTs). Their detailed functions have been reviewed by *Kruppa* et al. [35]. As such, the mitochondrial organization depends highly on the cytoskeleton, which allows mitochondrial redistribution and plays a key role in the immune response. Activated T-helper (Th) cells that form contact with other cells within the immune response (e.g., antigen-presenting cells, MSCs) show mitochondrial movement towards the interaction sites [36]. Here, the higher mitochondrial density is suggested to ensure Ca^2+^ influx and thereby to maintain Th-cell activation. The modulatory effect of MSCs on immune cells (T-cells, natural killer (NK)-cells, or macrophages) has been reported recently [[37], [38], [39], [40]]. Besides, it has been shown that the components of the actin-cytoskeleton directly contribute to mitochondrial function.

**Fig. 2:**
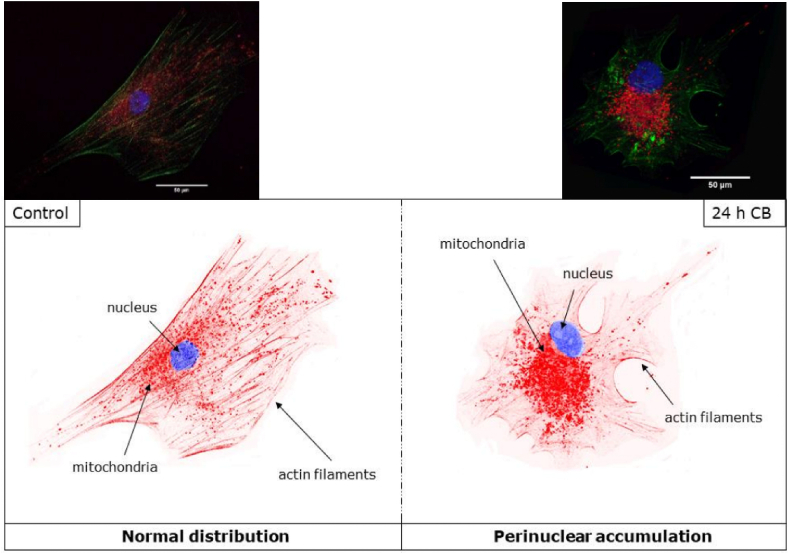
**Effect of actin-based cytoskeleton disruption on mitochondrial distribution.** Control MSCs show a normal mitochondrial distribution (red) with minimal perinuclear accumulation (nucleus in blue). Treatment of MSCs for 24 h with 16 μM cytochalasin B (24 h CB) triggers actin filament depolymerization, which results in increased perinuclear accumulation of mitochondria.

Treatment of MSCs with cytochalasin B (CB), which is a cell-permeable mycotoxin that affects the filamentous actin-based cytoskeleton network formation, results in an impaired mitochondrial network, shown by a perinuclear accumulation of mitochondria caused by abnormal fission and fusion dynamics (Fig. 2) [41]. Mitochondrial mass and volume in these cells decrease compared to healthy cells; notably, the mitochondrial volume of CB-treated MSCs increases compared to total cell volume. These effects could be reversed during a 24 h-recovery phase, demonstrating the highly regenerative abilities of MSCs [41].

### 2.3 Mitochondrial transfer

**Fig. 3:**
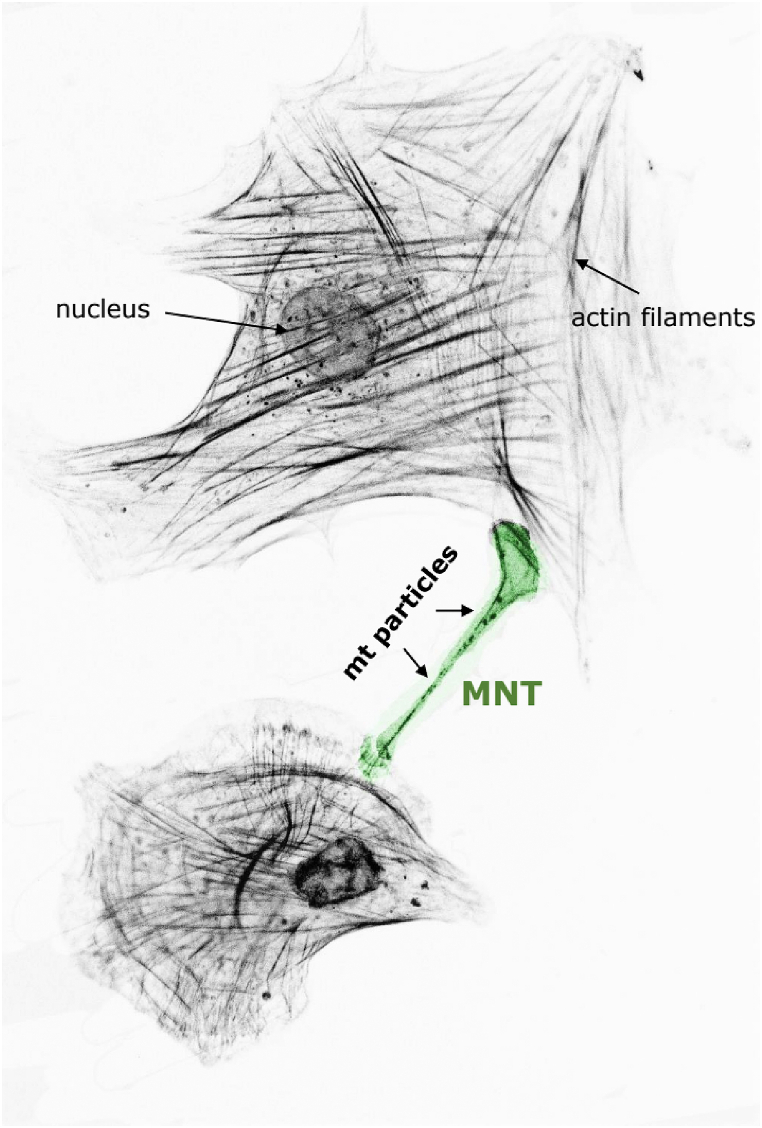
**Mitochondrial transport through nanotunnels by MSCs.** Mitochondrial nanotunnels (MNTs, green) are tubular structures containing mostly of F-actin that can be formed by MSCs to transport mitochondrial particles or other cellular components (e.g., extracellular vesicles) to other MSCs or other cell types (e.g., endothelial cells, platelets) to restore their vitality and support their function, preventing apoptosis.

Beside to the intracellular exchange of signaling molecules that affect cellular function, MSCs can transfer whole mitochondria or mitochondrial DNA to other cells to restore their health and ensure their functionality [42,43]. Automatic quantification of mitochondrial transfer in a large scale is challenging. There are some methods available to analyze mitochondrial movement in living cells, but they are inadequate for high-throughput analyses [44,45]. Microfluidic chambers in combination with the MitoQuant software have been used to successfully examine mitochondrial transport in living neurons [46]. This software tool allows the analysis of large, high-resolution data sets in a time-efficient manner and could be adapted for other cellular models, e.g., the transfer of mitochondria through nanotunnels. For the directed transfer of mitochondria, double-membraned, tubular protrusions of <1–30 μm length, so-called mitochondrial nanotunnels (MNTs), can be formed (Fig. 3) [47]. These structures are built through a coordinated network of cytoskeletal structures (MTs, microfilaments) for the transfer of mitochondria, but also for lysosomes or extracellular vesicle exchange. It has been suggested that they are formed by deficient cells to serve as a rescue signal; however, that might not always be the case. MSCs, might act as cellular “paramedics” using nanotunnels to donate their intact mitochondria to restore mitochondrial function in other cells. It has been shown that mitochondrial transfer can extend the life span of the recipient cells [48,49] and is involved in the immunomodulatory function of MSCs [50].

There is an increasing demand for precise analysis tools that can deal with large imaging data sets to measure mitochondria in living cells and the mitochondrial movement through specialized structures in a dynamic 3D system.

## 3 State-of-the-art technologies to analyze mitochondrial physiology

Mitochondrial morphology and matrix content are good indicators for the (patho-)physiological state of a cell. Thus, accurate experimental procedures to measure mitochondrial dimensions are required for the assessment of alterations. Here, state-of-the-art imaging techniques will be discussed to visualize mitochondria that are compatible with microfluidic devices*.* These detection systems are divided into off-chip systems, in which the detection parts are not integrated within the microfluidic device, or on-chips providing fully integrated imaging systems. However, some optical techniques are difficult to miniaturize or loose sensitivity due to shortened optical path lengths.

**Light scattering** has been widely used in the past, but only allows the measurement of mitochondrial dimensions in pre-isolated mitochondria [51,52]. Intracellular characterization using common extraction processes lacks sufficient functionality of the organelle and the complex reticular network they form within the cell.

Dyes to detect mitochondrial proteins or the mitochondrial membrane potential (MMP) are often used in **microscopic analysis** of mitochondrial volume; notably, mitochondrial proteins could be targeted by certain pathophysiological processes and longer incubation times for detection of mitochondrial potential leads to ROS formation. Small alterations in the mitochondrial volume, e.g., due to cell contraction, would not be detectable either. Besides, microscopic methods show a limited spatial resolution and in case of objects at or below the diffraction limit, artificial enlargement of the objects through diffraction blur occurs. Hence, these objects appear considerably thicker, and the mitochondrial volume would be overestimated. This effect also depends on the shape of the object, as a rod-shaped form would be more affected than a spherical, leading to inconsistent data. Recent adaptations on confocal microscopy improved the resolution below the diffraction limit (e.g., combination of confocal and deconvolution microscopy). Here, correctly calibrated deconvolution allows the removal of diffraction-induced blur; thus, the actual size of the mitochondrion is visible.

**Electron microscopy** provides the necessary resolution for accurate analysis; however, the cells need to be fixed, which affects mitochondrial volume and excludes the observation of functional processes. Recently, a new approach for volume measurement based on microscopic analysis has been published [53]. Here, laser diffraction is used to measure the length of the sarcomere, if through tight packing one sarcomere encompasses two mitochondria. Through light pacing it is even possible to use this technique to quantify dynamic changes in mitochondrial volume *in situ*. However, this approach is only suitable for short observation times and many variables can influence the analysis, from cell manipulation (contraction or stretching) to the size of the analyzed cell population.

The most common method for optical analysis of mitochondria using microfluidics is **fluorescence microscopy,** permitting the observation and evaluation of mitochondria in fixed cells or live-cell-imaging of mitochondria. However, autofluorescence of certain polymers or biomolecules would affect the efficiency of the detection. Modern fluorophore-coupled probes (e.g., mitochondria-specific MitoTracker Red) are highly sensitive, allowing the precise detection of individual proteins, biological structures, or MMP of active mitochondria (tetramethylrhodamine ethyl ester -TMRE) using confocal microscopy or flow cytometry [54,55]. Besides, quenching or photobleaching can occur, influencing the intensity of the signal used for calculation [56,57]. In case of live-cell-imaging, the wavelength used for fluorophore excitation could harm the cells, especially in time-course studies where repeated examinations will be performed. Nanoengineered optical probes, such as quantum dots, nanoparticles, or biosensors, could provide a suitable alternative providing higher intensity and longevity and are ideal for multiplexed detection [58].

A variety of fluorescence-based methods can be used to determine mitochondrial damage. Changes in mitochondrial mass, activity, membrane potential or superoxide production can be determined by either confocal fluorescence microscopy or flow cytometry. Both methods are based on specific fluorescent antibodies for detection and allow a highly sensitive analysis of fixed or living cells in a dynamic or end-point setup requiring low cell amounts. Flow cytometry has the advantage of quantitatively determining the intracellular expression of single cells within a heterogenous population in a multiplexed assay. In contrast, fluorescence microscopy is limited in the use of different fluorescent dyes simultaneously. MitoSOX™ dyes are widely used for live-cell imaging to detect mitochondrial reactive oxygen species (ROS). Augmented production of ROS results in mitochondrial damage, including mutations in the mitochondrial DNA or damage to components of the respiratory chain. In case of using adherent cells, the potential of scraping or trypsinization to induce oxidative stress should be considered in the assay design. However, flow cytometry is still a powerful tool to analyze many samples in a short period of time providing large data sets on different parameters.

## 4 Microfluidic devices as promising technology to monitor mitochondrial function

**Fig. 4:**
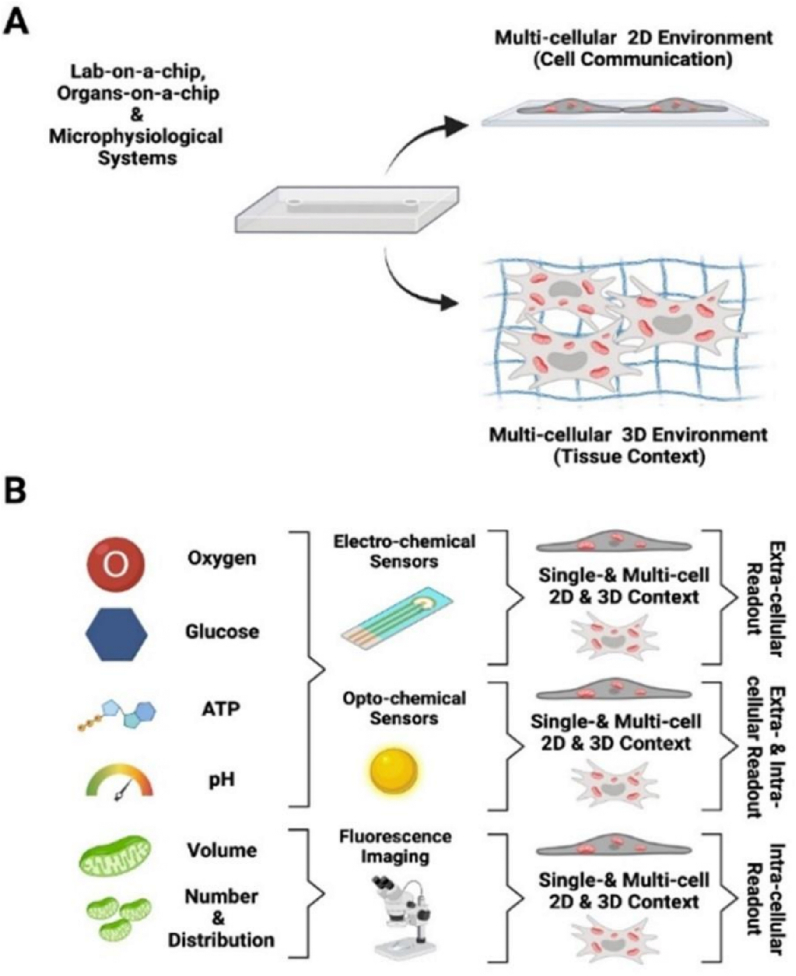
Overview of microfluidic technologies. Lab-on-a-chip, organ-on-a-chip and microphysiological systems A) with multi-cellular 2D and 3D environment to investigate cellular function, interaction, and tissue context. B) Oxygen and glucose consumption as well as ATP and pH-value measures can be determined using microfluidic devices.

Microfluidic devices, including organ- and lab-on-a-chip as well as MPS platforms are high-performance tools for drug discovery, disease diagnosis and (clinical) sample monitoring. They allow the analysis of small sample volumes, from microliter to picoliter, on dimensions of 10–100 μm, and various devices even incorporate valves and pumps for automated fluidic handling. Oxygen supply, an important cultivation parameter to maintain cellular physiology, can be controlled within microfluidic systems either by supplementation of soluble oxygen scavengers [59,60] to the culture medium or oxygen control microchannels next to the cultivation compartment exploiting the diffusion of nitrogen and dissolved oxygen [61] from the donor channel via diffusion through a gas permeable barrier. Further, microfluidic chips can be fabricated from materials with oxygen scavenging properties, e.g., thiol-containing photopolymers, such as OSTEmer, using flow rate adjustments or optimizations in the post-baking temperature of biochips to adjust oxygen scavenging rates [62]. Another straight-forward approach for oxygen control is to leverage the natural respiration of the cells within the tissue construct to deplete oxygen within biochips made from gas-impermeable material and tune the oxygen gradients using fluid perfusion control [63]. From an analytical perspective, the combination of oxygen monitoring approaches with other vital metabolic microprobes and sensors can give rise to analytical platforms capable of precise investigation of mitochondrial metabolic (dys-)function in the context of 3D cell culture models (Fig. 4). Metabolic measurements by the Agilent Seahorse Bioanalyzer emerged over the past years and has become a valuable tool to measure mitochondrial function and glycolysis in living cells. The assay can be used for cells, organoids, tissue slices and fresh patient samples, but requires specific culture plates.

*Bavli* et al. [64] established a real-time mitochondrial respiration approach using two tissue-embedded phosphorescent microprobes with combined electrochemical measurements of glucose and lactate to analyze shifts from oxidative phosphorylation to anaerobic glycolysis, an early indicator of mitochondrial stress. Microfluidic devices offer a comprehensive, versatile landscape to study real-time and *in situ* mitochondrial function on transcriptional, translational, and epigenetic level in individual cells in response to environmental changes, showing an excellent compatibility with optical detection methods. Microfluidic-integrated electro-chemical biosensors can monitor variations in oxygen demand, pH-values, and ROS production enabling a detailed metabolic analysis of the mitochondrial performance [63].

Mitochondrial functions of individual MSCs can be evaluated in a more realistic, tissue-like setup [[65], [66], [67]]. A disbalance in the mitochondrial redox system during MSC cultivation for MSC-based therapeutic use has a major impact on treatment efficiency, as shown previously [68]. Furthermore, novel therapeutic modalities to increase the MMP using photo-biomodulation were applied by Ref. [69]. The combination of microfluidic-hydrostatic trap arrays with fluorescent staining can be used effectively to improve single-cell-analysis of mitochondrial homeostasis when employing probes to measure MMP by TMRE [70]. MSC interactions with other cells have been investigated to correlate deficient actin-cytoskeleton function with a disturbance in oxygen, emphasizing the importance of 2D and 3D cultivation techniques for the functional performance of the cell [41]. Using microfluidic chips fabricated with optical sensor spots of oxygen-sensitive microparticles, oxygen pressure and consumption of MSCs could be assessed under dynamic conditions.

Alternatively, oxygen demand and medium acidification (an indicator for mitochondrial deficiency) can be measured in parallel using integrated opto-chemical microbead-sensors. Fluorescence-based analysis systems using opto-chemical sensors can apply fluorescent dyes to analyze mitochondrial function and energy metabolism of MSCs within 3D culturing systems, while microbead probes are more suitable to analyze cell metabolism within bulk 3D tissue models. Considering their biodegradability, magnetization, and fluorescence, nanoparticles (e.g., metal colloids, quantum dots and liposomes) or nanoprobes, such as oxygen nanoprobes, that can target mitochondria offer benefits of integration into microfluidic devices for the investigation of intracellular oxygen and glucose metabolisms. However, surface modifications on a microscale level remain challenging. Glass, oxidized silicon, and polymers are widely used showing the advantages of easily produced hydrophilic surfaces at low costs. To address the increasing demand of reproducible, disposable high-throughput microfluidic devices that meet the geometrical accuracy for clinical settings, improvements are required. Further, the short duration of the hydrophilic effect in some cases can be problematic. Grafting techniques could overcome this problem but are not yet suitable for mass production. 3D printing technologies provide a promising alternative to chemical and physical device fabrication [[71], [72], [73]]. Widely used materials for 3D printing of microfluidic devices are stereolithography (SLA) [74,75], digital light projection (DLP) [76,77], fused deposition modeling (FDM) [78,79] and direct ink writing (DIW) [80,81]. Photosensitive polymers, including SLA and DLP, show some limitations in designing thin channels (blockage of tunnels with material remnants) [82]. FDM and DIW offer a wide repertoire of materials accessible with high biocompatibility (e.g., polylactic acid) [83,84] and transparency optimal for optical detection methods. Recently, Dowsil 732 from Dow Corning [85] has been reported as suitable material for microfluidic devices. The material provides hydrophilic, transparent surfaces and the production of the devices is cost- and time-efficient, making them suitable for clinical applications.

## 5 Computational analysis of microfluidic data

Capturing the dynamics of gene and protein expression and their regulation in complex systems in response to their environment is challenging. Combination of optical approaches with microfluidic devices holds promise for a broad spectrum of application, including quantitative methods based on fluorescent markers, co-localization in a tissue-specific environment, and variations in populations. Recently, computational fluid dynamics (CFD) has been widely used in the analyses of fluid flow, mixing, and chemical reactions in microfluidic devices to improve flow rate and device design. The software makes it possible to simulate the blood flow in models for vascular diseases [86].

The most common computational methods for processing and analysis of imaging data are listed in Table 1. ImageJ-Fiji [87] is the most widely used platform for mitochondrial imaging analysis. The open-source software allows an automated 2D analysis of mitochondrial morphology, number (Mitochondrial Morphology macro), and distribution (MitoLoc) through microscopic analysis of single image layers [88]. For the calculation of mitochondrial volume based on z-stacks, 3D models were developed. These algorithms include the definition of mitochondrial volume and shape as well as network analysis. These open-source packages for ImageJ-Fiji [87], named Mitochondrial Analyzer and Mitochondrial Network Analysis (MiNA) [89] are user-friendly workflows for 3D analysis of mitochondrial dynamics.

**Table 1:** Overview of different software features compatible with microfluidic data to determine mitochondrial dynamics.

Software	Feature	URL	Description	Application
*ImageJ-Fiji*		https://imagej.net/imagej-wiki-static/Fiji		
	MitoLoc		Problem with elongated structures; structural clustering	Accurate structural illustration; detection of shape differences; mitochondrial number and distribution
	Particle Analyzer		Auto-threshold; inaccurate analysis of elongated structures (more mitochondria detected than present); structural clustering	Accurate structural illustration; mitochondrial number; detection of shape differences; mitochondrial area
	Mitochondrial Morphology		Problem with elongated structures; structural clustering	Accurate structural illustration; detection of shape differences;
	MiNA		Processing of large data sets; no extensive structural clustering; 3D viewer; high resolution z-stacks required; 2D/3D skeleton or Ridge Detection plugin for segmentation	Accurate determination of mitochondrial shape, distribution, area and volume; network analysis; branching; analysis of small and large structures; network analysis; accurate structural illustration; clearly distinguishes shape differences; mitochondrial number
	Mitochondrial Analyzer		Adaptive-threshold (improved structural segmentation); no extensive structural clustering; deconvolution improves recognition but decreases accuracy to detect small objects; processing of large data sets; 3D viewer; high-resolution z-stacks required; 2D/3D skeleton or Ridge Detection plugin for network analysis	Accurate determination of mitochondrial shape, distribution, area and volume; network analysis; analysis of small and large structures; network analysis; accurate structural illustration; clearly distinguishes shape differences; mitochondrial number
*MatLab-MitoQuant*		https://doi.org/10.1007/s13238-016-0268-3		
	MitoTracker		Processing of large data sets	Localization of mitochondria in 3D-images; mitochondrial number
	Motion Pattern Analyzer		Processing of large data sets; uses trajectories generated by MiTracker; 3D trajectory map	Mitochondrial movement; transient velocity analysis (mobility and net movement)
*MatLab-Stys algorithm*		https://github.com/jl8kii/conv-mt	3D-visualization; Gaussian-based segmentation; Gaussian blur; no post-filtering necessary; high resolution z-stacks required; 3D analysis	Accurate determination of mitochondrial shape, distribution, area and volume; accurate structural illustration, mitochondrial number

To calculate the mitochondrial area, which is referred to as mitochondrial footprint in MiNA, and volume, the binarized images are analyzed for the number of positive signals (pixels/voxels) multiplied by the total area/volume (pixels/voxels). Mitochondrial morphology can be described by the number of punctuates, rods and networks per cell. Network analysis is accomplished by the 2D/3D Skeleton or Ridge Detection plugins. The Ridge Detection plugin requires additional parameters, including high contrast, low contrast, line width, and minimum length. However, both rely on well-resolved, high-contrast images with no or at least minimal structural overlap. For visualization of the mitochondrial shape and distribution ImageJ-Fiji provides a 3D viewer based on Java 3D which generates a 3D visualization of the image z-stack, including volumes and surfaces.

The main technical task in the analysis process is segmentation, either through binary images [90] or machine learning [91]. The first relies on an empirically set threshold but makes no assumption on shape and structure of the observed object, the latter assumes the characteristics of an object based on a priori reference data, which is disadvantageous in case of anomalies, like changes in shape or excessive aggregation. The Matlab-based algorithm provided by the lab of Dalibor Stys (Institute of Physical Biology, University of South Bohemia) allows automated processing of the data combined with the least assumption of the object parameters (available here https://github.com/jl8kii/conv-mt). Because of their radially symmetric shape, mitochondria are approximated as ellipsoid [92] and used Gaussian-based segmentation as followed. By segmenting all ellipsoid-looking local maxima, no post-filtering of the results is necessary. It calculates different anisotropies of density, and then filters out relatively radial isotropic ones (single objects without branching). The main advantage of this model is the transparency and the absence of implicit bias since all limits are explicitly set, making it suitable for the statistical analysis of individual objects.

Recently, the Matlab-based toolkit “MitoQuant” for the analysis of mitochondrial movement has been provided [46] for the specific analysis of large imaging data sets generated using microfluidic devices. It consists of the program “MiTracker” for the localization of mitochondria by linking their coordinates into 3D trajectories and the motion pattern analyzer to determine mitochondrial movement.

The combination of automated programs with the optical analysis of microfluidics offers great potential to examine MSC vitality before their therapeutic use and to study the role of biological processes in MSCs in human diseases associated with mitochondrial dysfunction.

## 6 Clinical application of mitochondrial analysis using microfluidic-based technologies

Due to the heterogeneity of mitochondria and remaining challenges in automated, high-throughput mitochondrial analysis, the use of microfluidic systems are promising tools for clinical research to improve diagnosis of pathologies associated with mitochondrial dysfunction. Compatible high-throughput microfluidic-based technologies enable to study the mitochondrial heterogeneity among patients, tissues, and even individual cells.

Defective mitochondrial homeostasis is associated with a variety of human diseases. The chronic low-grade systemic inflammation in metabolic syndrome (MetS), co-occurring with obesity, hypertension, and insulin resistance, is associated with oxidative stress in MSCs of the adipose tissue. Upon MetS the protein transport into MSC mitochondria is impaired, resulting in low energy levels [93]. To a certain degree, mitoquinone (MitoQ), a mitochondrial targeted antioxidant, can counteract MetS conditions by reducing mitochondrial oxidative stress and increasing mitophagy and mitochondrial biogenesis [94]. However, the decreased energy requirements in MetS reduce PGC-1α levels, contributing to ROS toxification and alterations in mitochondrial mass and function [95]. Such key regulators can serve as markers for disease prediction. The two cytoplasmic microRNAs (miRNAs) miR-15a-5p and miR-17–5p can modulate mitochondrial function, leading to a decrease in ATP production and ROS formation and, thus, have been postulated as targets for MetS [96].

The mitochondrial physiology in MSCs can serve as quality control for their use as therapeutic agents. Besides, mitochondria could act as effective targets to regulate MSC’ functionality and differentiation status in clinical applications. Microfluidic devices provide high flexibility and reproducibility to develop flow systems showing complex chamber geometries and features that mimic the anatomy and physiology of the cellular environment, e.g., the design of a microvascular system to create an *in vitro* model for vascular diseases [97]. These devices allow both, the control of the biochemical and biophysical conditions, and the continuous generation of real-time data that can be transformed into clinical knowledge.

## 7 Conclusion

Monitoring of MSCs for their use in therapeutics benefits from small sample volume, accuracy, and the continuous transmission of real-time data comprising the morphological and biochemical status of single cells. Microfluidic-based technologies are widely used in cellular biosensors and enable highly accurate modeling of cell-cell or cell-substrate interactions. Future development of microfluidic platforms using an electrochemical detection system to directly measure mitochondrial function on the level of electronic transfer or ATP generation. The metabolism of MSCs is dynamic and highly compartmentalized, thus, technical approaches that allow the visualization of subcellular ATP will be of interest for various research fields. A continuous integration of innovative technologies into microfluidic systems can substitute for animal testing and accomplish dynamic monitoring of critical MSC parameters in real time with consistent conditions to generate precise and reproducible data. Considering their potential to address challenges in clinics regarding sample collection, preparation and analysis, microfluidic platforms can improve the predictive power of functional assays and reduce incidence of failure in late-stage clinical trials, thereby reducing costs and speeding up developmental progress of cell-based therapies.

## 8 Summary

Mitochondrial volume homeostasis is essential for maintaining the structural integrity of the organelle. Mitochondrial dimensions and functionality are correlated with the energy demand of the cell and can be used as markers for diseases affecting mitochondrial function. Therefore, it is important to improve experimental and computational techniques to ensure an appropriate correlation between mitochondrial analysis and disease phenotypes. Analysis of mitochondrial network, cellular distribution, and mitochondrial functionality could give rise to a diagnostic tool for human diseases. Mitochondria are highly dynamic structures, and their rapid morphological and molecular adaptations to environmental changes complicate their precise analysis. Furthermore, cell-specific properties, e.g., of MSCs which can exchange their mitochondrial content, either through MNT or EV formation, should be considered, e.g., as an indicator for tissue repair. Beside mitochondrial volume, the relation of mitochondrial volume to cell volume, and their shape and distribution could be crucial parameters for diagnostics. In this context the current approaches for the determination of mitochondrial dimensions and functionality are challenged by their limitation to reflect the actual (patho-)physiological status of a living cell.

The advances in high-throughput microfluidic devices for morphological and molecular mitochondrial analysis are promising to address the increasing demand of accurate, disposable, reproducible applications, e.g., for *in vitro* diagnostic devices and health screenings. While glass and polymers are the most prominent materials used for microfluidic devices, 3D printing technologies offer new possibilities for specific applications to medical analysis in the future.

In combination with a compatible cellular model, such as MSCs, 3D mitochondrial analysis of live-cell images might offer a diagnostic tool for diseases characterized by mitochondrial dysfunction. Monitoring clinical samples benefits from small sample volume, accuracy, and the continuous transmission of real-time data comprising the morphological and biochemical status of individual cells.

## Authors contribution

SS wrote the manuscript and MBF and PE revised it. SS and MR designed the figures using Biorender. KL and DS wrote the Matlab-Stys algorithm. All authors approved the final draft of the manuscript.

## Funding statement

Dalibor Stys was supported by Ministerstvo Školství, Mládeže a Tělovýchovy [LM2018099], European Regional Development Fund [GAJU 017/2016/Z].

Michael Fischer was supported by Interreg [AT-CZ 215].
